# Supplementary material for: Endoplasmic Reticulum Stress Promotes the Expression of TNF-α in THP-1 Cells by Mechanisms Involving ROS/CHOP/HIF-1α and MAPK/NF-κB Pathways
Source: Int J Mol Sci. 2023 Oct 14;24(20):15186. doi: 10.3390/ijms242015186 (PMC10606873; doi:10.3390/ijms242015186)
Supplement: Supplementary file 1 [file ijms-24-15186-s001.zip › ijms-2538896-supplementary.pdf]

Supplementary Figures: S1 to S4

Manuscript titled: Endoplasmic Reticulum Stress Promotes the Expression of TNF-α in THP-1 Cells by Mechanisms Involving ROS/CHOP/HIF-1α and MAPK/NF-κB Pathways

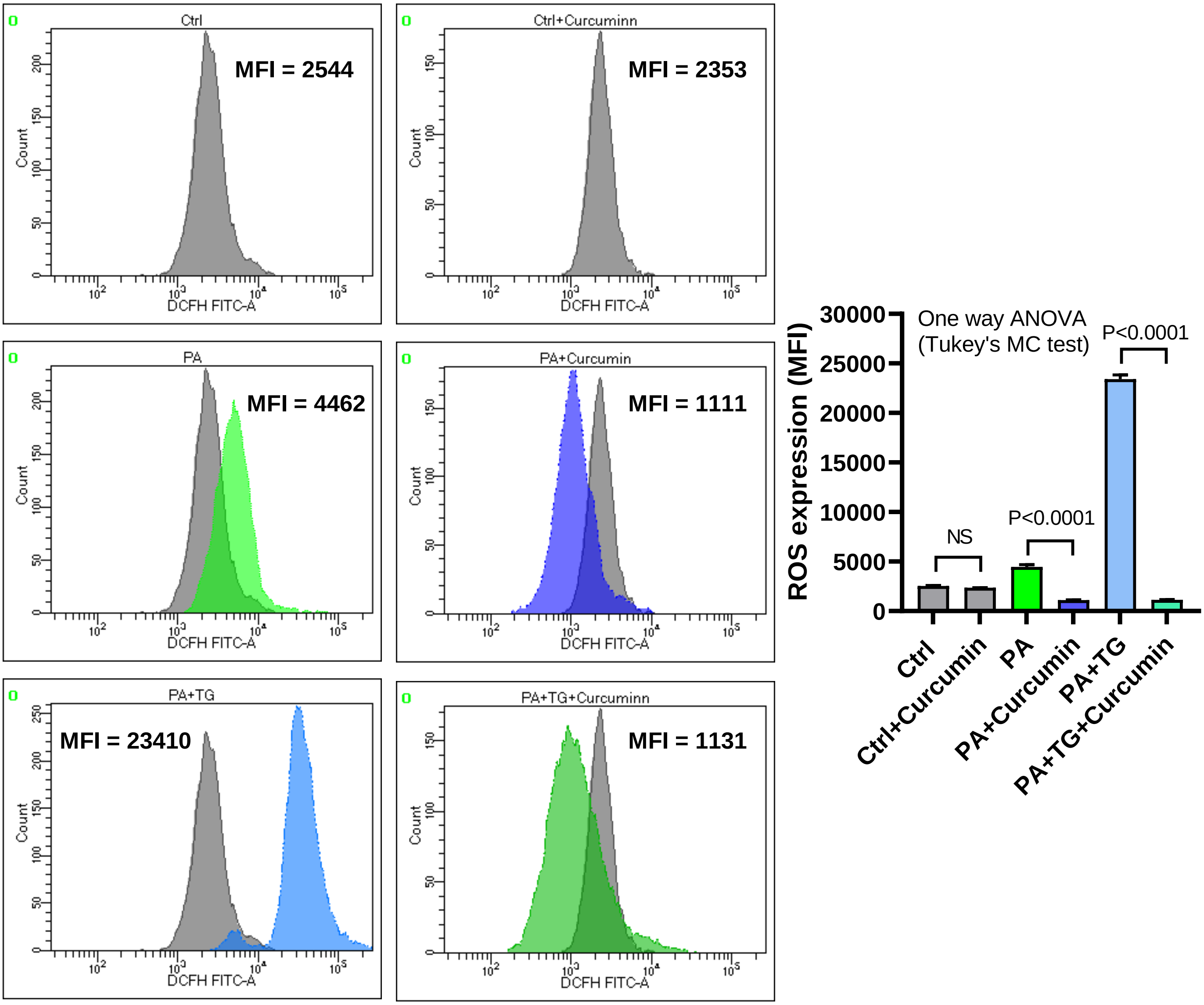

Supplementary Figure S1. Changes in intracellular reactive oxygen species (ROS) are oxidation dependent. THP-1 cells were plated at a cell density of 1×10^6 cells/mL/well in triplicate wells of 12-well plates and the cells in designated wells were pre-treated (1h) with curcumin and then stimulated with palmitate (PA, 200 µM) and with palmitate (PA, 200 µM) + Thapsigargin (TG, 1 µM), including vehicle (0.1% BSA) treatment as control, and cells were incubated at 37°C for 24h. Intracellular ROS was measured using DCFH-DA assay and flow cytometry as described in Materials and Methods. Similar results were obtained from three independent experiments and flow cytometry data (expressed as mean±SEM) representing ROS expression were analyzed using One-way ANOVA, Tukey's multiple comparisons test. $P<0.05$ was considered significant. The histogram and graph data show significant suppression of ROS when cells were stimulated after treatment with curcumin, suggesting that ROS expression was oxidation dependent and not affected by influx/efflux of DCFH probe by lipid stimulant.

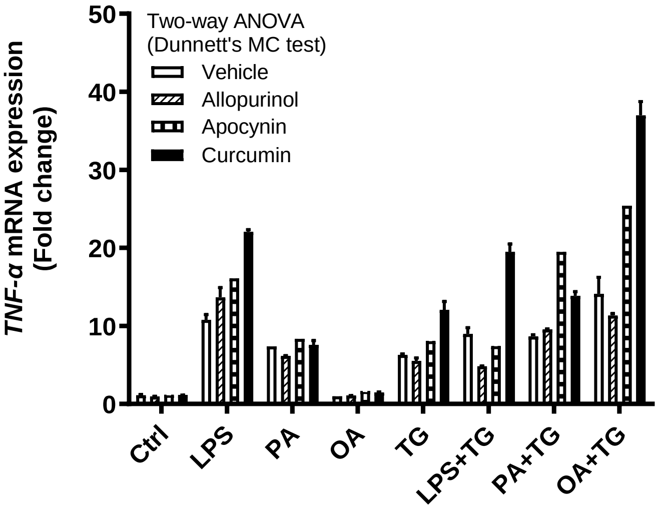

Supplementary Figure S2. Effect of antioxidants/ROS scavengers on *TNF-α* gene expression. THP-1 cells were plated at a cell density of 1×10^6 cells/mL/well in triplicate wells of 12-well plates and the cells in designated wells were pre-treated (1h) with antioxidants/ ROS scavengers including allopurinol, apocynin, and curcumin and then stimulated with lipopolysaccharide (LPS, 10 ng/mL), palmitate (PA, 200 μM), and oleate (OA, 200 μM), in presence or absence of the ER stressor thapsigargin (TG, 1 μM), while anti-oxidant control wells were pre-treated with vehicle (0.1% BSA) and later stimulated likewise other cells that were pre-treated with antioxidants. After 24h incubation (37ºC), cells were harvested, lysed in RLT buffer, total RNA was purified and *TNF-α* gene expression was determined using qRT-PCR as described in Materials and Methods. Similar results were obtained from three independent experiments. Data (expressed as mean±SEM) were analyzed using two-way ANOVA, Dunnett’s multiple comparisons test. The representative data from 3 independent experiments with similar results show that, compared with respective vehicle control for each condition, no antioxidant or ROS scavenger suppressed the expression of *TNF-α* at the transcriptional level. On the contrary, an increase in *TNF-α* mRNA levels was observed following priming with these agents.

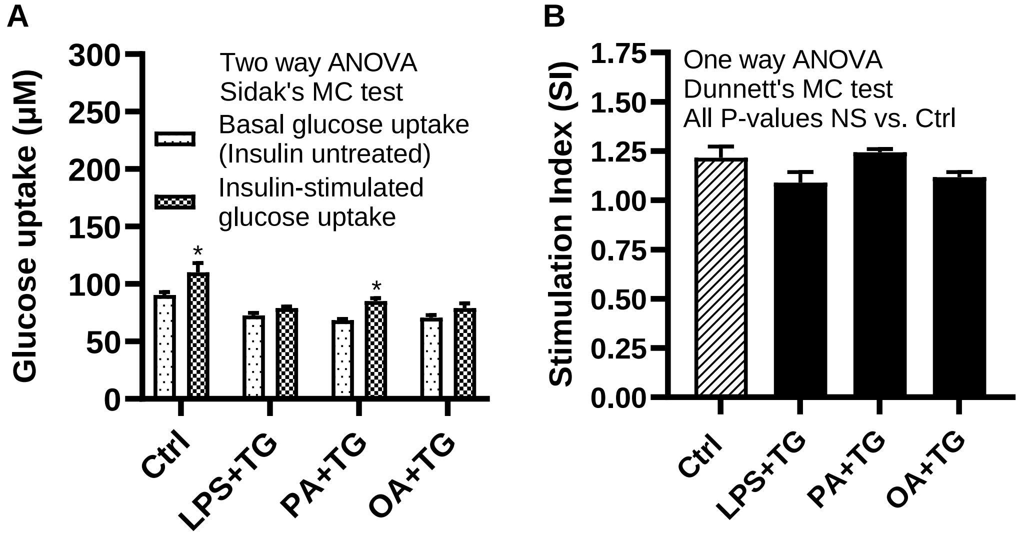

Supplementary Figure S3. Effect of combination treatments on glucose uptake in THP-1 cells. THP-1 cells were plated at a cell density of 1×10^6 cells/mL/well in triplicate wells of 12-well plates and stimulated with LPS (10 ng/mL), PA (200 µM), and OA (200 µM), in presence of TG (1 µM), while control was treated with vehicle (0.1% BSA) only and incubated for 24h. Cells were washed, replated, serum starved for 18h, and seeded in plates (5×10^4 cells/100 µL/aliquot) for treatments (in duplicate) including: (1) sample background control; (2) insulin stimulated cells; and (3) non-insulin stimulated control. Cells were glucose starved by pre-incubation with 100 µL KRPH buffer (2% BSA) for 40 min. Background control was washed 3× but 2-deoxyglucose (2-DG, a glucose analog) was not added. Insulin stimulated cells were incubated with 2 µM insulin in KRPH buffer (2% BSA) for 20 min to activate glucose transporters and then 10 mM 2-DG (10 µL) was added to both insulin stimulated cells and non-insulin stimulated control and incubated at 37° C for 20 min. The assay was carried out as described in Materials and Methods. Glucose uptake were calculated as follows: 2-DG uptake = (Ts/Sv) * D = pmol/µL = nmol/mL = µM; where, Ts = Amount of 2-DG6P in sample tube calculated from standard curve (pmol), Sv = Sample volume (µL), D = Sample dilution factor (if diluted for optimization) Representative data (mean±SEM) from 2 independent experiments with similar results are shown. Data were analyzed using two-way ANOVA (Dunnett's/ Sidak's multiple comparisons test) and P<0.05 was considered significant. (A) Insulin stimulation induced significant increase in glucose uptake in vehicle (Ctrl) or PA+TG treated cells only. (B) Stimulation index for glucose uptake was comparable between Ctrl and treatments, implying that these combination treatments did not affect the glucose uptake in THP-1 cells. * $p < 0.05$

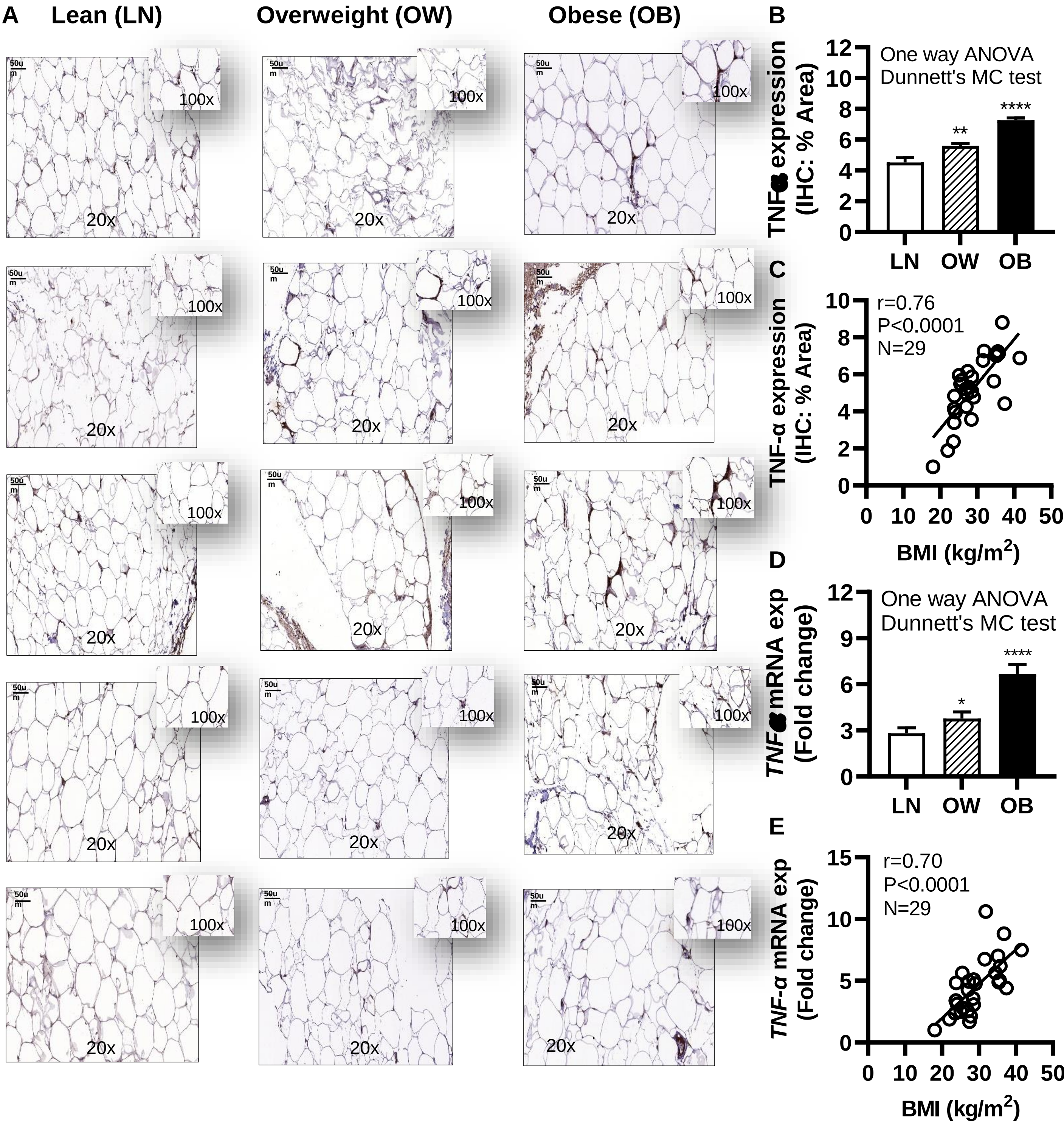

Supplementary Figure S4. TNF-*α* expression in the adipose tissue. Adipose tissue samples from 9 lean/LN (BMI 23.46±0.78 kg/m^2), 10 overweight/OW (BMI 27.66±0.45 kg/m^2), and 10 obese/OB (BMI 35.55±1.23 kg/m^2) individuals (cohort 1), were collected by abdominal fat pad biopsy, TNF-*α* protein expression was assessed by immunohistochemistry (IHC) and *TNF-α* gene expression using RT-qPCR as described in Materials & Methods. (A) The representative IHC images from 3 independent stainings with similar results, 5 individuals each, are shown (20× magnification, scale bar 50 μm). Insets are shown at 100× magnification. (B) Adipose TNF-*α* protein expression in OW and OB is compared with LN individuals. (C) Correlation between TNF-*α* protein expression and BMI. (D) *TNF-α* gene expression in OW and OB is compared with LN individuals. (E) Correlation between *TNF-α* gene expression and BMI. * $p < 0.05$; ** $p < 0.01$; **** $p < 0.0001$

Supplementary Tables: S1 and S2

Manuscript Title: Endoplasmic Reticulum Stress Promotes the Expression of TNF-α in THP-1 Cells by Mechanisms Involving ROS/CHOP/HIF-1α and MAPK/NF-κB Pathways

Supplementary Table S1. Characteristics of Study Population (Cohort 1)

Parameter	Lean	Overweight	Obese
Age (Yrs)	45.28±4.92	43.30±5.51	43.70±6.07
Gender (Male/Female)	5/4	6/4	5/5
BMI (kg/m^2)	23.46±0.78	27.66±0.45	35.55±1.23
Blood glucose (mmol/L)	5.09±0.23	5.24±0.33	5.11±0.27
TC (mmol/L)	5.45±1.04	5.08±0.37	5.38±0.51
HDL-c (mmol/L)	1.49±0.30	1.16±0.13	1.14±0.13
LDL-c (mmol/L)	3.22±0.75	3.21±0.35	3.78±0.45
TG (mmol/L)	0.49±0.04	1.55±0.31	1.06±0.19

BMI: Body mass index; TC: Total cholesterol; HDL-c: High-density lipoprotein cholesterol; LDL-c: Low-density lipoprotein cholesterol; TG: Triglycerides

Supplementary Table S2. Characteristics of Study Population (Cohort 2)

Parameter	Lean	Overweight	Obese
**Age (Yrs)**	42.20±3.80	36.84±5.06	36.67±5.48
**Gender (Male/Female)**	7/5	6/6	5/7
**BMI (kg/m^2)**	21.69±0.56	27.71±0.38	39.51±2.17
**Blood glucose (mmol/L)**	5.32±0.20	5.16±0.30	5.26±0.19
**TC (mmol/L)**	4.91±0.82	5.64±0.53	4.62±0.44
**HDL-c (mmol/L)**	1.47±0.32	1.37±0.18	1.08±0.14
**LDL-c (mmol/L)**	3.30±0.55	3.25±0.40	3.40±0.36
**TG (mmol/L)**	0.70±0.13	1.30±0.29	1.13±0.19
**TNF-α (pg/mL)**	7.35±1.43	8.55±2.53	14.94±2.49

BMI: Body mass index; TC: Total cholesterol; HDL-c: High-density lipoprotein cholesterol; LDL-c: Low-density lipoprotein cholesterol; TG: Triglycerides; TNF-α: Tumor necrosis factor-α
